# Elevated resting heart rate is associated with increased radiographic severity of knee but not hand joints

**DOI:** 10.1038/s41598-021-03237-4

**Published:** 2021-12-08

**Authors:** Sung-Eun Choi, Haimuzi Xu, Ji-Hyoun Kang, Dong-Jin Park, Sun-Seog Kweon, Young-Hoon Lee, Hye-Yeon Kim, Jung-Kil Lee, Min-Ho Shin, Shin-Seok Lee

**Affiliations:** 1 Division of Rheumatology, Department of Internal Medicine, Chonnam National University Medical School & Hospital, 42 Jebong-ro, Dong-gu, Gwangju, 61469 Republic of Korea; 2 Department of Preventive Medicine, Chonnam National University Medical School, 160 Baekseo-ro, Dong-gu, Gwangju, 61469 Republic of Korea; 3 Jeonnam Regional Cancer Center, Chonnam National University Hwasun Hospital, Hwasun, Republic of Korea; 4 Department of Preventive Medicine and Institute of Wonkwang Medical Science, Wonkwang University College of Medicine, Iksan, Republic of Korea; 5 Gwangju-Jeonnam Regional Cardiocerebrovascular Center, Chonnam National University Hospital, Gwangju, Republic of Korea; 6 Department of Neurosurgery, Chonnam National University Medical School and Hospital, Gwangju, Republic of Korea

**Keywords:** Diseases, Rheumatology

## Abstract

Although the resting heart rate (RHR) predicts the clinical outcomes of cardiovascular disease, chronic obstructive lung disease, diabetes mellitus, and the risk of cancer, its role in patients with musculoskeletal diseases, such as osteoarthritis (OA), remains unclear. We explored the association of the RHR with the extents of radiographic changes in the knees and hands of 2369 subjects from the Dong-gu Study. The radiographic hand and knee joint findings were graded semi-quantitatively; we calculated total hand and knee joint scores. Multiple linear regression was performed to examine the associations between the RHR and the radiographic characteristics of these joints. For the knee joints, the RHR was associated positively with the total (*p* < 0.01), osteophyte (*p* < 0.01), joint space narrowing (JSN; *p* < 0.01), and tibial attrition (*p* = 0.02) scores after adjustment for age, sex, body mass index, smoking status, alcohol consumption, educational and physical activity levels, and comorbidities. For the hand joints, the RHR was associated positively with the JSN (*p* = 0.01) and subchondral cyst (*p* < 0.01) scores after such adjustment. The RHR was not associated with the total, osteophyte, sclerosis, erosion, or malalignment score for the hand joints. This study is the first to reveal an association between the RHR and the radiographic severity of knee, but not hand, OA.

## Introduction

Osteoarthritis (OA) is a common disabling condition occurring in the appendicular joints of the knees, hips, and hands. OA damages the articular cartilage and triggers osteophyte formation and synovial inflammation. Patients with this disease experience joint pain, stiffness, instability, and limitations of motion that reduce their quality of life and induce functional limitations^1^. In the Fifth Korean National Health and Nutrition Examination Survey (2010–2012), 9.3% of males and 28.5% of females exhibited symptomatic OA, and 16.4% of males and 23.9% of females exhibited radiographic OA^2^. OA is associated significantly with major healthcare costs, greatly increasing household economic burdens^3^.

The heart rate reflects the oxygen demand of the myocardium, blood flow in the coronary arteries, and myocardial performance. In general populations, low resting heart rates (RHRs) are associated with good health and longevity, and high RHRs are associated with cardiovascular diseases, chronic obstructive lung disease, diabetes mellitus, and the risk of cancer^4–7^. A few studies have explored the relationship between the RHR and musculoskeletal diseases, including rheumatoid arthritis (RA). RA is characterized by increases in systemic inflammation^8^ and sympathetic activity^9^, and elevated RHRs are associated with increased levels of inflammatory markers such as high-sensitivity C-reactive protein (hsCRP), and sympathetic overactivation. It would thus be intuitive to expect that the RHR may be elevated in RA patients compared to healthy individuals. In one study, the RHR has been found to be elevated in patients with RA relative to healthy controls^10^, and associated with more organ damage^11^. In another study, however, the RHRs of patients with RA and controls did not differ^12^. Little is known about the relationship between the RHR and musculoskeletal diseases such as OA. Thus, we investigated the associations of the RHR with the radiographic characteristics of knee and hand OA in a large population-based cohort.

## Methods

### Population and study design

We enrolled subjects from the Dong-gu Study, a population-based cohort study conducted from 2007 to 2010 in the Dong-gu area of Gwangju Metropolitan City^13^. This was a cross-sectional study that formed part of a larger cohort study and the relationship between the RHR and OA radiographic severity was investigated at one time point. Of 9260 subjects aged ≥ 50 years, 2516 underwent imaging to explore OA development and progression in 2009. In total, 2489 subjects who underwent knee and hand joint x-ray imaging were screened for inclusion in the present study. Fifty-one subjects were excluded because they had histories of knee amputation or total knee replacement. We also excluded 8 subjects for whom alcohol consumption data were lacking, eight subjects for whom comorbidity data were missing, 23 subjects for whom RHR data were lacking, and 38 subjects with atrial fibrillation. Finally, 2369 subjects were enrolled. The Institutional Review Board of Chonnam National University Hospital approved this study (no. CNUH-2021-092) and all patients provided written informed consent at the time of enrollment. This study adhered to the Declaration of Helsinki and the Good Clinical Practice guidelines.

### Covariates

We used questionnaires to collect sociodemographic data, including data on the subjects’ age, sex, educational and physical activity levels, and smoking and alcohol consumption statuses. Body mass indices (BMIs) were calculated as the weight (in kilograms) divided by the height (in meters) squared. Subjects’ educational levels were classified as high school or above. Physical activity was classified as moderate or vigorous. Moderate activity was performed for at least 30 min five times per week and included doubles tennis, volleyball, badminton, table tennis, swimming, yoga, and toning exercises that slightly increase the heart rate. Vigorous physical activity was performed for at least 20 min three times per week, and included running, mountain-climbing, soccer, basketball, rope-jumping, singles tennis, and squash. The subjects were classified as never-smokers (< 100 lifetime cigarettes and currently not smoking), ex-smokers (> 100 lifetime cigarettes and currently not smoking), and current smokers (> 100 lifetime cigarettes and currently smoking). Their alcohol consumption in the past 12 months was used to distinguish current alcohol consumers from non-consumers. Hypertension was defined as a systolic blood pressure ≥ 140 mmHg and/or a diastolic blood pressure ≥ 90 mmHg on ≥ 2 occasions, and/or self-reported antihypertensive medication use. Diabetes mellitus was defined as a fasting glucose level ≥ 140 mg/dL or the use of insulin or a hypoglycemic agent. Dyslipidemia was considered present when a subject was taking any lipid-lowering medication. In addition, we checked for any history of coronary heart disease, cerebrovascular disease, chronic liver disease, chronic pulmonary disease, or cancer. The RHRs were measured, radiographic imaging was performed, and the questionnaires were completed in the morning of the same day.

### Heart rate measurement

The RHR was measured with each subject supine after 5 min rest using a 12-lead electrocardiogram (PageWriter 200 M1771A; Hewlett Packard, MA, USA). Subjects with atrial fibrillation were excluded. The RHRs were divided into quartiles: 35–57, 58–63, 64–69, and 70–126 bpm.

### Radiographic scoring

Anteroposterior X-rays of the hand and knee joints (four limbs) were obtained with each subject standing. Two trained rheumatologists working independently scored the images using a semi-quantitative method by reference to the Atlas of Standard Radiographs^14^. The total OA score was the sum of the scores for the various radiographic features. Intra- and inter-observer reliability were tested using data from a subgroup of participants. The intraobserver (κ = 0.85–0.92) and interobserver (κ = 0.79–0.89) reliabilities were good, as reported previously^15^. The semi-quantitative grading of knee OA yielded a maximum score of 42. The maximal osteophyte, joint space narrowing (JSN), tibial attrition, and sclerosis scores are 24, 12, 4, and 2, respectively. For the hand joints, the maximum score is 70 points and the maximum osteophyte, JSN, subchondral cyst, sclerosis, erosion, and malalignment scores are 22, 22, 4, 6, 10, and 6, respectively.

### Statistical analysis

Data processing and statistical analyses were performed using STATA version 14.2 (StatCorp., Austin, TX, USA). General characteristics are presented as means with standard deviations (SDs) for continuous variables and as numbers with percentages for categorical variables. Continuous variables were compared by one-way analysis of variance, and categorical variables were compared using the chi-squared test. The relationships between the RHR and OA radiographic characteristics were explored by multiple linear regression after adjustment for confounding factors including age, sex, BMI, smoking and alcohol consumption status, educational and physical activity levels, and comorbidities (hypertension, diabetes mellitus, dyslipidemia, coronary heart disease, cerebrovascular disease, chronic liver disease, chronic pulmonary disease, and cancer). In addition, the Bonferroni correction was used to adjust the comparisons between each RHR quartile and the individual knee and hand OA radiographic scores. We performed logistic regression analyses to explore whether higher RHRs reflected the median total radiographic scores for the knee and hand joints. *P* values < 0.05 were taken to indicate significance.

## Results

The baseline characteristics and radiographic features of the knee and hand joints are shown by the RHR in Table 1. The mean age of the 2369 subjects was 64.0 years (SD = 8.3 years) and the mean BMI was 24.4 kg/m^2^ (SD = 2.9 kg/m^2^); 1030 (43.5%) subjects were male. At the time of evaluation, we collected data on pain severity. Subjects were asked if they had experienced any knee or hand joint pain in the past month, and 111 of 2328 (4.77%) subjects reported knee joint pain and 397 of 2344 (16.94%) reported hand joint pain. In terms of comorbidities, 830 (35.0%) subjects had hypertension and 314 (13.3%) had diabetes mellitus. Subjects in the highest RHR quartile were older (p < 0.01), more likely to be male (p < 0.01), less likely to be current alcohol consumers (p = 0.04), less educated (p = 0.04), and less physically active (p = 0.01) than those in the lower quartiles. Subjects in the highest RHR quartile were more likely to have hypertension (p = 0.01) and diabetes mellitus (p < 0.01) than those in the lower quartiles. The rates of other comorbidities, including dyslipidemia, coronary heart disease, cerebrovascular disease, chronic respiratory disease, liver disease, and cancer, did not differ among the RHR quartiles.

**Table 1:** Baseline characteristics of study participants according to the RHR (beats/min).

	Total	Quartile 1 (35–57)	Quartile 2 (58–63)	Quartile 3 (64–69)	Quartile 4 (70–126)	*P*-value
Number	2,369 (100.0)	625 (26.4)	601 (25.4)	566 (23.9)	577 (24.4)	
Age, years	64.0 ± 8.3	63.6 ± 8.3	63.6 ± 8.0	63.4 ± 8.1	65.5 ± 8.5	< 0.01
BMI, kg/m^2^	24.4 ± 2.9	24.2 ± 2.8	24.4 ± 2.7	24.6 ± 2.9	24.5 ± 3.2	0.10
Males (%)	1030 (43.5)	303 (48.5)	250 (41.6)	203 (35.9)	274 (47.5)	< 0.01
Current smokers (%)	292 (12.3)	74 (11.8)	71 (11.8)	60 (10.6)	87 (15.1)	0.12
Current alcohol consumers (%)	1182 (49.9)	338 (54.1)	305 (50.7)	264 (46.6)	275 (47.7)	0.04
Education, above high school (%)	766 (32.3)	226 (36.2)	198 (32.9)	162 (28.6)	180 (31.2)	0.04
Physical activity, moderate or vigorous (%)	384 (16.2)	127 (20.3)	85 (14.1)	92 (16.3)	80 (13.9)	0.01
Hypertension (%)	830 (35.0)	188 (30.1)	215 (35.8)	197 (34.8)	230 (39.9)	0.01
Diabetes mellitus (%)	314 (13.3)	51 (8.2)	64 (10.6)	71 (12.5)	128 (22.2)	< 0.01
Dyslipidemia (%)	201 (8.5)	47 (7.5)	51 (8.5)	56 (9.9)	47 (8.1)	0.52
Coronary heart disease (%)	128 (5.4)	32 (5.1)	34 (5.7)	25 (4.4)	37 (6.4)	0.49
Cerebrovascular disease (%)	105 (4.4)	25 (4.0)	26 (4.3)	24 (4.2)	30 (5.2)	0.77
Chronic respiratory disease (%)	153 (6.5)	33 (5.3)	36 (6.0)	35 (6.2)	49 (8.5)	0.13
Liver disease (%)	447 (18.9)	106 (17.0)	118 (19.6)	100 (17.7)	123 (21.3)	0.21
Cancer (%)	106 (4.5)	27 (4.3)	27 (4.5)	21 (3.7)	31 (5.4)	0.59
**Knee joints**						
Total score	14.53 ± 6.91	13.84 ± 6.76	14.29 ± 6.58	14.16 ± 6.57	15.91 ± 7.52	< 0.01
Osteophyte score	7.65 ± 3.74	7.32 ± 3.66	7.63 ± 3.56	7.36 ± 3.55	8.30 ± 4.12	< 0.01
JSN score	6.05 ± 2.56	5.73 ± 2.55	5.89 ± 2.45	6.03 ± 2.53	6.58 ± 2.62	< 0.01
Tibial attrition score	0.23 ± 0.58	0.22 ± 0.57	0.20 ± 0.54	0.19 ± 0.50	0.32 ± 0.67	< 0.01
Sclerosis score	0.60 ± 0.92	0.56 ± 0.91	0.56 ± 0.92	0.58 ± 0.87	0.70 ± 0.99	0.03
**Hand joints**						
Total score	16.90 ± 6.26	16.42 ± 5.82	16.68 ± 6.03	16.67 ± 6.08	17.90 ± 6.98	< 0.01
Osteophyte score	6.49 ± 2.12	6.51 ± 2.15	6.48 ± 2.05	6.40 ± 2.09	6.57 ± 2.21	0.58
JSN score	8.58 ± 3.30	8.24 ± 3.23	8.45 ± 3.17	8.49 ± 3.17	9.16 ± 3.55	< 0.01
Subchondral cyst score	1.02 ± 1.26	0.90 ± 1.21	0.94 ± 1.23	1.02 ± 1.23	1.25 ± 1.33	< 0.01
Sclerosis score	0.39 ± 0.85	0.41 ± 0.88	0.39 ± 0.81	0.38 ± 0.85	0.40 ± 0.87	0.91
Erosion score	0.28 ± 0.97	0.25 ± 0.88	0.29 ± 1.00	0.25 ± 0.90	0.36 ± 1.08	0.20
Malalignment score	0.13 ± 0.49	0.11 ± 0.44	0.13 ± 0.50	0.12 ± 0.47	0.17 ± 0.53	0.17

Unless otherwise indicated, data are shown as means ± standard deviations.

*RHR* resting heart rate, *BMI* body mass index, *JSN* joint space narrowing.

In terms of knee-joint radiographic features, subjects in the higher RHR quartiles had significantly higher total (p < 0.01), osteophyte (p < 0.01), JSN (p < 0.01), tibial attrition (p < 0.01), and sclerosis (p = 0.03) scores than those in the lower quartiles. In terms of hand-joint radiographic features, subjects in the higher RHR quartiles had significantly higher total (p < 0.01), JSN (p < 0.01), and subchondral cyst (p < 0.01) scores than those in the lower quartiles. The hand-joint osteophyte, sclerosis, erosion, and malalignment scores did not differ among the RHR quartiles.

Table 2 shows the relationships between RHR and total and individual radiographic scores for the knee and hand joints. In multiple linear regression analyses adjusted for age, sex, BMI, smoking and alcohol consumption status, educational and physical activity levels, and comorbidities (including hypertension, diabetes mellitus, dyslipidemia, coronary heart disease, cerebrovascular disease, chronic respiratory disease, liver disease, and cancer), the RHR was positively associated with the total (p < 0.01), osteophyte (p < 0.01), JSN (p < 0.01), and tibial attrition (p = 0.02) scores for the knee joints, and with the JSN (p = 0.01) and subchondral cyst (p < 0.01) scores for the hand joints. When the four quartiles were directly compared after Bonferroni correction, RHR quartile 4 had a significantly higher overall radiographic score, total (p < 0.01), osteophyte (p = 0.02), and JSN (p < 0.01) scores for the knee joints, and JSN (p = 0.01) and subchondral cyst (p < 0.01) scores for the hand joints than RHR quartile 1. When we analyzed the association between the RHR and sum of the total radiographic scores of the knee and hand joints the RHR was significantly associated with the total radiographic scores of the knee and hand joints after adjustment (p < 0.01).

**Table 2:** Association of RHR (beats/min) quartiles with the total and individual radiographic scores of the knee and hand joints.

	Quartile 1 (35–57)	Quartile 2 (58–63)	Quartile 3 (64–69)	Quartile 4 (70–126)	*P*-value	*P*-value* (Q2 vs. Q1)	*P*-value* (Q3 vs. Q1)	*P*-value* (Q4 vs. Q1)
**Knee joints**								
Total score	14.21 ± 0.24	14.41 ± 0.25	14.12 ± 0.25	15.42 ± 0.25	< 0.01	1.00	1.00	< 0.01
Osteophyte score	7.51 ± 0.14	7.69 ± 0.14	7.32 ± 0.14	8.07 ± 0.14	< 0.01	1.00	1.00	0.02
JSN score	5.86 ± 0.09	5.94 ± 0.09	6.03 ± 0.1	6.40 ± 0.10	< 0.01	1.00	0.54	< 0.01
Tibial attrition score	0.24 ± 0.02	0.21 ± 0.02	0.19 ± 0.02	0.29 ± 0.02	0.02	0.92	0.23	0.52
Sclerosis score	0.59 ± 0.04	0.57 ± 0.04	0.58 ± 0.04	0.66 ± 0.04	0.37	1.00	1.00	0.65
**Hand joints**								
Total score	16.75 ± 0.21	16.8 ± 0.21	16.77 ± 0.22	17.31 ± 0.22	0.20	1.00	1.00	0.19
Osteophyte score	6.53 ± 0.08	6.53 ± 0.08	6.48 ± 0.08	6.42 ± 0.08	0.75	1.00	1.00	1
JSN score	8.43 ± 0.11	8.49 ± 0.11	8.49 ± 0.12	8.91 ± 0.12	0.01	1.00	1.00	0.01
Subchondral cyst score	0.97 ± 0.04	0.95 ± 0.04	1.00 ± 0.04	1.17 ± 0.04	< 0.01	1.00	1.00	< 0.01
Sclerosis score	0.43 ± 0.03	0.40 ± 0.03	0.40 ± 0.03	0.35 ± 0.03	0.44	1.00	1.00	0.31
Erosion score	0.27 ± 0.04	0.30 ± 0.04	0.26 ± 0.04	0.31 ± 0.04	0.79	1.00	1.00	1
Malalignment score	0.12 ± 0.02	0.13 ± 0.02	0.12 ± 0.02	0.15 ± 0.02	0.64	1.00	1.00	0.69
Knee and hand joint total score joints	30.96 ± 0.38	31.21 ± 0.39	30.89 ± 0.40	32.73 ± 0.40	< 0.01	1.00	1.00	< 0.01

Data are shown as means with standard errors, derived via multiple linear regression adjusted for age, gender, body mass index, smoking and alcohol consumption status, educational and physical activity levels, and comorbidities (hypertension, diabetes mellitus, dyslipidemia, coronary heart disease, cerebrovascular disease, chronic respiratory disease, liver disease, and cancer).

*RHR* resting heart rate, *JSN* joint space narrowing, *Q* quartile.

*Bonferroni-corrected *P*-value (for all three tests).

Table 3 shows the relationships between RHR levels above and below the median and the total radiographic scores for the knee and hand joints above and below the median. In multivariable logistic regression analyses adjusted for age, sex, BMI, smoking and alcohol consumption status, educational and physical activity levels, and comorbidities (hypertension, diabetes mellitus, dyslipidemia, coronary heart disease, cerebrovascular disease, chronic respiratory disease, liver disease, and cancer), subjects with RHRs above the median had higher total radiographic scores for the knee joint (p < 0.01) but not the hand joint (p = 0.48). In addition, subjects with RHRs above the median had higher summed total radiographic scores for the knee and hand joints in multivariable logistic regression analyses after adjustment (p = 0.01).

**Table 3:** Multivariable logistic regression analysis testing whether RHR values above the median were associated with the total median radiographic scores of the knee and hand joints.

	Odds ratio (95% confidence interval)	*P*-value
Knee joint total score*		
RHR (low vs. high)	1.52 (1.23–1.88)	< 0.01
Age (years)	1.11 (1.1–1.13)	< 0.01
Gender (male vs. female)	1.23 (0.99–1.54)	0.06
Body mass index (kg/m^2^)	1.12 (1.08–1.15)	< 0.01
Current smoker (yes vs. no)	1.21 (0.9–1.63)	0.20
Current alcohol consumer (yes vs. no)	0.92 (0.76–1.11)	0.39
Education, above high school (yes vs. no)	0.65 (0.53–0.8)	< 0.01
Physical activity, moderate or vigorous (yes vs. no)	0.85 (0.67–1.09)	0.20
Hypertension (%)	1.04 (0.85–1.27)	0.73
Diabetes mellitus (%)	0.78 (0.59–1.03)	0.08
Dyslipidemia (%)	1.26 (0.91–1.74)	0.17
Coronary heart disease (%)	0.74 (0.49–1.1)	0.13
Cerebrovascular disease (%)	1.57 (0.99–2.49)	0.05
Chronic respiratory disease (%)	1.05 (0.73–1.52)	0.79
Liver disease (%)	1.04 (0.82–1.31)	0.77
Cancer (%)	0.99 (0.65–1.51)	0.96
Hand joint total score*		
RHR (low vs. high)	1.08 (0.87–1.35)	0.48
Age (years)	1.16 (1.14–1.17)	< 0.01
Gender (male vs. female)	1.84 (1.46–2.32)	< 0.01
Body mass index (kg/m^2^)	1.03 (1–1.07)	0.06
Current smoker (yes vs. no)	1.08 (0.8–1.47)	0.62
Current alcohol consumer (yes vs. no)	1.11 (0.91–1.36)	0.29
Education, above high school (yes vs. no)	0.86 (0.7–1.06)	0.15
Physical activity, moderate or vigorous (yes vs. no)	0.79 (0.61–1.02)	0.07
Hypertension (%)	1.18 (0.95–1.45)	0.13
Diabetes mellitus (%)	0.94 (0.7–1.25)	0.66
Dyslipidemia (%)	1.05 (0.75–1.47)	0.77
Coronary heart disease (%)	1.01 (0.67–1.53)	0.95
Cerebrovascular disease (%)	1.26 (0.79–2.03)	0.34
Chronic respiratory disease (%)	1.19 (0.8–1.75)	0.40
Liver disease (%)	1.14 (0.89–1.44)	0.30
Cancer (%)	1.03 (0.66–1.6)	0.90
Knee and hand joint total score*		
RHR (low vs. high)	1.35 (1.07–1.69)	0.01
Age (years)	1.16 (1.14–1.18)	< 0.01
Gender (male vs. female)	1.09 (1.05–1.12)	< 0.01
Body mass index (kg/m^2^)	1.71 (1.35–2.16)	< 0.01
Current smoker (yes vs. no)	1.39 (1.02–1.9)	0.04
Current alcohol consumer (yes vs. no)	0.99 (0.81–1.22)	0.96
Education, above high school (yes vs. no)	0.62 (0.5–0.77)	< 0.01
Physical activity, moderate or vigorous (yes vs. no)	0.78 (0.6–1.01)	0.06
Hypertension (%)	1.11 (0.9–1.37)	0.34
Diabetes mellitus (%)	0.84 (0.63–1.12)	0.23
Dyslipidemia (%)	1.19 (0.85–1.68)	0.31
Coronary heart disease (%)	0.79 (0.52–1.2)	0.27
Cerebrovascular disease (%)	1.7 (1.04–2.79)	0.03
Chronic respiratory disease (%)	1.11 (0.74–1.64)	0.62
Liver disease (%)	1.13 (0.89–1.44)	0.32
Cancer (%)	1.40 (0.89–2.18)	0.14

*RHR* resting heart rate.

*The total scores of the knee and hand joints were dichotomized based on median split.

## Discussion

In this study, we found that the RHR was associated with the total, osteophyte, JSN, and tibial attrition scores for the knee joints, and with the JSN and subchondral cyst scores for the hand joints in multiple linear regression after adjustment. When the RHRs and total radiographic scores of the knee and hand joints were dichotomized for multivariable logistic analyses, an elevated RHR was associated with increased radiographic severity of the knee, but not the hand joint. To the best of our knowledge, this is the first study to identify an association between the RHR and radiographic severity of OA using a semi-quantitative grading system in a large population-based cohort.

In this investigation, we found that elevated RHRs were associated with the increased radiographic severity of knee OA. Elevated RHRs are known to be associated with higher levels of inflammatory biomarkers, including high-sensitivity C-reactive protein, interleukin-6, and fibrinogen, reflecting sympathetic overactivation and mechanical stressing of the vascular endothelium^16^. As substantial evidence suggests that subclinical inflammation is linked to OA development, we postulate that chronic low-grade inflammation mediates the association between the RHR and OA radiographic severity. Interestingly, an exploratory analysis of the Osteoarthritis Initiative cohort showed that the use of beta blockers (heart rate–reducing agents) was associated with less radiographic progression than was non-use^17^. In the Genetics of Osteoarthritis and Lifestyle study, beta-blocker use was associated with less joint pain and opioid analgesic use among patients with symptomatic knee or hip OA^18^. Although no study has investigated the relationship between the RHR and OA severity directly, the effect of beta blockers suggests that RHR elevation is linked to increased OA symptomatic and radiographic severity. This study was cross sectional; a longitudinal study is required to establish the relationship between the RHR and radiographic OA progression.

Notably, we found no association between the RHR and the radiographic severity of hand OA. In our previous study of the Dong-gu cohort, body weight was associated positively with the radiographic severity of OA in the knee, but not hand, joints^19^. Similarly, the serum adiponectin level was associated positively with the radiographic severity of knee, but not hand, OA^20^. These findings were explained by obesity and differences in the expression levels of adiponectin receptors. Indeed, the relationship between physical activity and symptoms and severity of knee OA appears to be bidirectional^21^. In general, physical activity reduces pain, and improves physical function and health-related quality of life among patients with knee OA compared to less physically active OA. However, more severe pain caused by worsening knee OA limits physical activity and thus compromises cardiovascular fitness^22^, elevating the RHR. Hand pain limits physical activity to a lesser extent than equivalent knee pain; the RHR is thus less likely to increase. As knee and hand pain may affect the extent of physical activity differently, the RHR would also be expected to be differentially affected. In addition, although further studies are needed to explore the different effects of the RHR on knee and hand joints, we postulate that a sympathetic neurotransmitter-induced adrenergic receptor signaling pathway exerts different effects on affected joints^23^. Radiographic OA severity varies according to the adrenergic receptor subtype and the levels of sympathetic nervous system neurotransmitters (especially norepinephrine), which affect knee and hand OA to different extents^24^.

The strengths of this study are the inclusion of a large number of subjects from a well-defined population, the use of a semi-quantitative scoring system for radiographic grading, and concurrent investigation of the knee and hand joints. However, our work has several limitations. First, as with any cross-sectional study, we could not establish a causal relationship between the RHR and radiographic OA severity. A longitudinal study is needed to examine causality. Second, as the RHR is affected by physical fitness, emotional stress, hormonal status, food, drugs, illness, and interactions between genetics and the environment^25^, residual confounding effects of unmeasured variables may have caused bias. Third, the heart rate exhibits diurnal variation; a single measurement of the RHR is not as good as multiple measurements, perhaps biasing the results. Fourth, although self-report questionnaires are commonly used to assess physical fitness, they vary in terms of what they measure (e.g., the mode, duration, and frequency of physical activity), how the data are reported (e.g., as activity scores, times, or calories burnt), the data quality (e.g., measures of intensity, differentiation of habitual from only recent activities; inclusion of measures of leisure and non-leisure activities) and how the data are obtained (e.g., paper and pencil assessment, computerized questionnaire, or via interview). The advantages of self-report questionnaires include cost-effectiveness, ease of administration, accuracy in terms of measurement of intense activity, and reliable classification of activity levels (e.g., low, moderate, or high). However, self-report questionnaires are less robust when used to measure light or moderate activity, or energy expenditure. Also, responses may be affected by the manner in which the questions are phrased, the perceived social desirability of exercise, questionnaire complexity, subject age, and the season in which the questionnaire is completed. Thus, bias may exist in estimates of associations between the RHR and OA radiographic severity.

## Conclusion

In this population-based study, RHR elevation was associated with the increased radiographic severity of knee, but not hand, OA. This study is the first to demonstrate an association between the RHR and radiographic OA severity. To further clarify the association between the RHR and OA radiographic severity, we plan to conduct a longitudinal follow-up study.

## Data Availability

The full study protocol and dataset can be accessed by academic researchers. Please contact Professor Shin-Seok Lee (shinseok@chonnam.ac.kr).
